# Naloxone availability in independent community pharmacies in Georgia, 2019

**DOI:** 10.1186/s13011-021-00402-w

**Published:** 2021-08-21

**Authors:** Lauren Gilbert, Jennifer Elliott, Lauren Beasley, Ekene Oranu, Kimberly Roth, Jennifer Nguyễn

**Affiliations:** 1 University of Houston College of Medicine, Houston, TX USA; 2 Mercer University College of Pharmacy, 3001 Mercer University Drive, Atlanta, GA USA; 3 Mercer University School of Medicine, Savannah, GA USA

**Keywords:** Naloxone, Pharmacies, Community pharmacy services, Opioids

## Abstract

**Background:**

Increasing the availability of naloxone among people who use opioids, and friends and family of past and present people who use opioids is a vitally important mission to reduce the occurrence of opioid-related overdose deaths. The purpose of this study was to determine the availability of naloxone in independent community pharmacies in Georgia. Secondary objectives include determining pharmacists’ knowledge regarding the standing order and ability to counsel regarding naloxone.

**Methods:**

A cross-sectional study using a secret shopper approach with phone contact was conducted over a period of 10 months. The study was population based and was conducted at all independent pharmacies in the state of Georgia. All independent community pharmacies in the state of Georgia were contacted and asked the naloxone questions with a 96% response rate (*n* = 520).

**Results:**

Five hundred fifty-eight independent community pharmacies were called, with a 96% response rate (n = 520 pharmacies). Two hundred-twenty pharmacies reported having naloxone in stock. Of the 335 pharmacists asked, 174 (51.9%) incorrectly said that a prescription was required. The mean (SD) cash price was $148.02 (27.40), with a range of $0 to $300. Of 237 pharmacists asked who had naloxone in stock or who stated they could get naloxone in stock, 212 stated that they could demonstrate how to use it, 8 stated they could not, and 17 said that they possibly could or were unsure how to use it.

**Conclusions:**

This study provided insight into the limited availability of naloxone at independent community pharmacies in Georgia after the standing order was issued. The majority of pharmacists at independent pharmacies in Georgia were not using the publicly available state naloxone standing order. Additionally, the low availability of naloxone and its high cost for uninsured individuals are significant structural barriers for reducing opioid-related mortality.

## Background

The opioid crisis claims more lives in the United States (US) each year [1]. Since 1999, nearly 841,000 people have died due to a drug overdose, with approximately 70% of these drug overdose deaths involving an opioid in 2019 [2]. The availability of naloxone, an opioid antagonist which rapidly reverses the effects of an opioid-induced respiratory depression, is a first line of defense in countering the incidence of opioid-related overdose deaths [3]. A single administration of a nasal spray or intramuscular injection of naloxone quickly competes for binding sites on opioid receptors within the nervous system, potentially saving an overdose victim’s life. Some people who use opioids may require a second or multiple doses of naloxone to reverse an overdose event [4]. Intranasal administration is an ideal alternative to the intramuscular injection route because it takes less skill for a layperson and there is no risk of a needlestick injury [3]. Having naloxone on hand is useful for people who take high doses of opioid pain medications, people who use opioid medications along with benzodiazepines, and people who use opioids not as prescribed by a doctor or illicit opioid medications or substances alike in order to reduce the risk of overdose mortality [5]. In 2017, then Surgeon General Jerome Adams encouraged widespread awareness of “knowing how to use naloxone and keeping it within reach [to] save a life” as a key part of the public health response to the opioid epidemic [6].

Increasing the availability of naloxone among people who use opioids, and friends and family of past and present people who use opioids is a vitally important mission to reduce the occurrence of opioid-related overdose deaths [5]. However, barriers to naloxone access are common due to issues such as expense [7], lack of awareness, lack of education [8], and stigma [9, 10]. Many studies have found that increased public awareness and increased pharmacist knowledge and training would be beneficial for increasing access to naloxone and potentially reducing opioid-related deaths [11–19]. Pharmacists are well positioned to identify patients on high opioid dosages who would benefit from having naloxone on hand, to dispense naloxone, and to counsel patients accurately on its usage and overdose signs and symptoms [10, 14, 15, 18, 20]. Recent policies passed by the American Pharmacists Association House of Delegates “supports access to third-party prescriptions for opioid reversal agents”; “supports the education for pharmacists … [on] the appropriate use of opioid reversal agents in overdose”; and “supports the pharmacist role in … providing education about the proper use of opioid reversal agents to prevention opioid-related deaths due to overdose.” [21]

All 50 states have mechanisms by which pharmacists can directly dispense naloxone to patients without a prescription from a physician, although specific access conditions vary by state [22–24]. Forty-two states as well as the District of Columbia allow the purchase of naloxone without the requirement of a prescription from a primary healthcare provider [5, 20]. A 2020 study found that adequate funding and staffing of local health departments and pharmacies, especially those in US counties with high rates of opioid overdose deaths, would be beneficial in supporting implementation of naloxone kit distribution and other opioid-related initiatives [8]. Studies in states that currently have standing orders, or orders in place that allow pharmacist to dispense naloxone to persons without prescriptions, have found a variety of availability of naloxone in retail community pharmacies. Previous studies have reported a wide range of the percentages of pharmacies contacted that reported having naloxone in stock, with some as low as 23.5% [25] and other as high as 97.7% [26] and many falling somewhere between [13, 14, 27–31].

Naloxone is available for purchase without a prescriber encounter in the state of Georgia under a standing order [32]. This standing order’s purpose is to “facilitate the widest possible availability of naloxone to ensure that family members, friends, co-workers, first responders, schools, harm reduction organizations and any other person or entities are in a position to provide assistance to a person experiencing an opioid related overdose through the timely administration of the opioid antagonist naloxone” [33]. The standing order for naloxone includes pre-filled syringes (nasal and intramuscular), intranasal liquids, intramuscular injection solutions, and auto-injectors. Previous research on naloxone access in Georgia examined a random sampling of pharmacies where the callers self-identified as researchers [34], and has explored naloxone access in rural community pharmacies [35, 36]. This project contributes a full census of independent community pharmacies in Georgia using a secret shopper method to mimic patient and provider interactions. This project aimed to assess the absolute and relative availability of naloxone in both urban and rural independent community pharmacies as well as pharmacists’ knowledge regarding the standing order and their ability to provide counsel on the medication.

## Methods

Mercer University’s Institutional Review Board deemed the study to be exempt for the need for informed consent because no patient data were used. Researchers obtained a list of licensed, community pharmacies from the Georgia State Board of Pharmacy and verified it for accuracy (i.e., to ensure no pharmacies were excluded, pharmacies were still in business, and verification of contact information) by conducting an internet and directory search of each zip code in the state. Independent pharmacies were defined as having fewer than four total locations [37]. The list was used to contact each independent community pharmacy. Trained researchers, including the authors and student researchers, used novel “secret shopper” methodology to call all independent pharmacies in Georgia posing as unidentified customers inquiring about the availability of naloxone at that pharmacy. This approach has been previously used in other studies accessing the availability of naloxone at pharmacies [31, 38]. One form of naloxone, a nasal spray, is FDA-approved and can be easily administered. The brand name for naloxone nasal spray, Narcan, was used to ask for the product as public recognition of the brand name is higher than the generic. Posing as patients (i.e. “secret shoppers”), researchers followed a script as they spoke to pharmacists to inquire about the availability of naloxone nasal spray at that location on the day the call was placed (Table 1). Because of dialect variation, we tried to ensure dialect concordance by having researchers with southern style accents call pharmacies located in counties whose residents are likely to have accents (i.e. counties that did not have urban or suburban cores). At the beginning of the call, researchers asked to speak with the pharmacist, and was insistent on speaking only to the pharmacist. If the pharmacist was not available, researchers asked for call backs or times the pharmacist will be available for the call. There was a maximum of three attempts for each pharmacy, with a one-week cool down period. If after three calls and the researchers could not speak to the pharmacist, they were not called again. During the call, if the pharmacist told the researcher that it was not in stock, the researcher asked where else it might be available. If the pharmacist stated that it was in stock, the researcher then asked for the retail price without insurance or any coupons applied, whether there was a less expensive alternative to the medication, and what the alternative’s price would be. Finally, researchers asked pharmacists if a prescription was required to obtain the medication. Data were collected and documented using Excel spreadsheet software version 2016 (Microsoft Corp) and SPSS statistical software version 25 (IBM Corp). Calls were placed between May 2019 and February 2020. Data analysis was performed June to July 2020.

**Table 1:** Telephone Responses - Accessibility of Narcan Under the Standing Order by Independent, Community Pharmacists in Georgia (*N* = 520)^a^

Questions	Responses, No./Total No. (%) [95% CI]
1. Do you have Narcan?	
a. Yes	220/520 (42.3)[38.0–46.7]
b. No	300/520 (57.7) [53.3–62.0]
2. If Narcan was not available … Can you tell me where I can find it?^+^	
a. Chain Store	130/300 (43.3) [37.7–49.2]
b. Vague Answer	77/300 (25.7) [20.8–31.0]
c. Unware of another pharmacy	52/300 (17.3) [13.2–22.1]
d. Other specific Store	20/300 (6.7) [4.1–10.1]
e. Offered to order product to the store	4/300 (1.3) [0.4–4.4]
f. Did not answer/hung up	17/300 (5.7) [3.3–8.9]
3. Can you show me how to use Narcan?^bc^	
a. Yes	212/237 (89.5) [84.8–93.1]
b. No	8/237 (3.4) [1.5–6.5]
c. Possibly could/unsure	17/237 (7.2) [4.2–11.2]
4. Do I need a prescription to get it?^d^	
a. Yes	173/334(51.8) [46.3–57.3]
B. No	135/334(40.4) [35.1–45.9]
c. Sometimes	11/3345(3.3) [1.7–5.8]
d. Unsure	15/334(4.5) [2.5–7.3]

^a^Specific questions asked to obtain answers 1–4: (1) “Do you guys have Narcan?” (2) If Narcan was not available, “Do you know where else I could find it?” (3) If pharmacist could demonstrate how to use Narcan, “Can you show me how to use it?” (4) “Do I need a prescription for that?”

^b^Not all pharmacists answered questions after reporting no Narcan in stock

^c^Despite not having Narcan currently in stock, some pharmacists offered to answer additional questions

^d^All pharmacists were asked this question, if possible

## Results

**Fig. 1:**
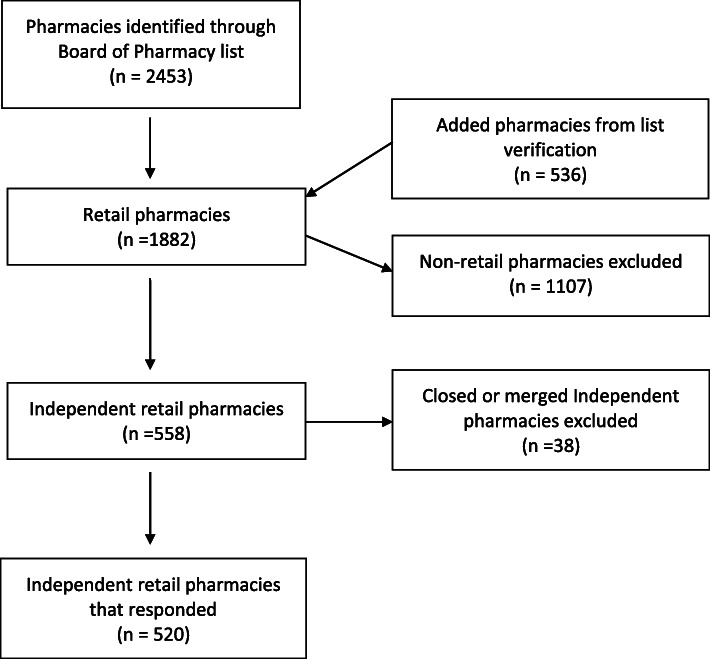
shows the flow of recruitment and inclusion and exclusion criteria

All 558 independent, community pharmacies were called, with a 96% response rate (*n* = 520 pharmacies; Fig. 1). Nonresponsive pharmacies included pharmacies that were going out of business (e.g. closing soon) or pharmacies that did not answer the call. Two hundred-twenty pharmacies (42.3%; 95% CI, 38.0–46.7%) reported having Narcan in stock (Table 1). Those pharmacists were then asked whether a prescription was necessary for purchase. Some pharmacists who did have Narcan stocked stayed on the phone so that further questions were able to be answered. Of the 335 pharmacists asked, 135 (40.3%; 95% CI, 35.0–45.8%) correctly responded in stating that a prescription was not necessary, 174 (51.9%; 95% CI, 46.4–57.4%) said that a prescription was required, 15 (4.5%; 95% CI, 2.5–7.3%) were unsure, and 11 (3.3%; 95% CI, 1.7–5.8%) said that a prescription was required in some situations. Of the 118 pharmacists who reported no Narcan in stock, 66 (55.9%; CI, 46.5–65.0%) also stated that individuals need a prescription to get Narcan. For those 216 pharmacists who reported having Narcan in stock, nearly half (49.5%; CI, 42.7–56.4%) also responded that a prescription was needed (Table 2). Some pharmacists who reported not having Narcan in stock still answered other questions in the guide, while others declined to answer additional questions.

**Table 2:** Comparison of prescription requirement response by Narcan availability

Question	Have Narcan in stock (Q1)	No Narcan in stock (Q1)
Do I need a prescription to get it? (Q3)		
a. Yes	107/216 (49.5) [42.7–56.4]	66/118 (55.9) [46.5–65.0]
b. No	99/216 (45.8) [39.1–52.7]	36/118 (30.5) [22.4–39.7]
c. Sometimes	4/216 (1.9) [0.5–4.7]	7/118 (5.9) [2.4–11.8]
d. Unsure	6/216 (2.8) [1.0–6.0]	9/118 (7.6) [3.6–14.0]

Pharmacists were also asked to give a cash quote for Narcan, meaning the cost without any discounts or insurance applied. The mean (standard deviation) cash price was $148.02 (27.40), with a range of $0 to $300. One pharmacist stated that their pharmacy does not charge for naloxone and will give it to anyone that requires the medication. Pharmacists were also asked if they could show the caller how to use the medication. Of 237 pharmacists asked who had naloxone in stock or who stated they could get naloxone in stock, 212 (89.5%; 95% CI, 84.8–93.1%) stated that they could, 8 (3.4%; 95% CI, 1.5–6.5%) stated they could not, and 17 (7.2%; 95%CI, 4.2–11.2%) said that they possibly could or were unsure how to do it. For pharmacists that reported that they did not have the medication in stock (*n* = 283, 54%), callers asked to be referred elsewhere to purchase the product. Of these, 130 (45.9%; 95% CI, 40.0–51.9%) specifically named a chain store; 77 (27.2%; 95% CI, 22.1–32.8%) gave a vague response such as “another nearby pharmacy should have it” or suggesting that the caller “call around”; 52 (18.4%; 95% CI, 14.0–23.4%) stated that they were unaware of another pharmacy that would have the medication in stock; 20 (7.1%; 95% CI, 4.4–10.7%) referred callers to another specific independent retail pharmacy; and 4 (1.4%; 95% CI, 0.4–3.6%) pharmacists offered to order the product to their store.

## Discussion

The results of the secret shopper study confirm that structural factors at the pharmacy level are a major barrier to increasing naloxone access and thus reducing mortality from the opioid epidemic in Georgia. The researchers found that, despite the standing order originally passed in January of 2017, less than half of independent community pharmacies in Georgia stock Narcan. Further, the results show that there is a considerable knowledge gap in regards to the naloxone standing order in Georgia: 59.5%of pharmacists incorrectly answering the question regarding obtaining naloxone without a prescription;10.5% of pharmacists in independent community pharmacies in Georgia do not feel comfortable providing counseling regarding naloxone’s use; and 43.0% of pharmacists gave vague answers or were unsure where a patient could obtain naloxone should they not have it in stock. Interestingly despite having the drug in stock, almost half of pharmacists still claimed that a prescription was required to dispense the drug (Table 2). These results underscore that provider under-education continues to be a barrier for adequate access to naloxone. Because of naloxone’s ability to save lives, it is imperative that pharmacists are made aware of its benefits and the laws surrounding its dispensing [5].

The richness of the data is generated by the fact that independent community pharmacies are not necessarily connected to one another; i.e., it is not likely to have multiple pharmacies respond in an extremely similar way. While a large chain may adhere to a corporate policy regarding the stocking and dispensing of Narcan and pharmacists are provided with standardized time sensitive training, there is generally no such widespread policy among independent pharmacies. Thus, calls to independent community pharmacies are better described as evaluations of pharmacists’ knowledge than of large corporations’ policies. Data are made still more robust by the variety in geographic location of the pharmacies.

Pharmacists who work in community settings are in an especially useful position in helping patients access naloxone as they are one of the most accessible healthcare professionals and may be able to identify patients using high dose equivalents of opioids who are good candidates for naloxone [39]. However, some independent community pharmacists display a lack of understanding of the proper counseling points associated with dispensing naloxone to people who use opioids [39, 40]. Furthermore, very few (1.4%) pharmacists spontaneously offered to order the prescription for the caller when asked where else to find the drug. While a small proportion, this willingness to secure the medication for potential patients highlights the importance of community pharmacists as gatekeepers for individuals who need access to the medication [36].

Pharmacists dispensing naloxone, with or without a prescription, should not assume that the patients have been given any proper training or counseling about its use [20]. Our study shows findings that having a naloxone standing order in place may not be as beneficial as intended given the lack of knowledge by the people who distribute the medication.

Additionally, cost may be a prohibitive factor for uninsured patients. The average cash price at the interviewed Georgia pharmacies currently stocking naloxone was $148.02, which is comparable to the national average of $150 [41]. As the average median annual household income in Georgia in 2018 is $55,679 [42], this may be viewed as a nonessential item for uninsured individuals in lower income brackets. This is particularly relevant, given that individuals who die by opioid overdose tend to be uninsured and of a lower socioeconomic status [43]. While people who use opioids who need access to naloxone come from all points of the socioeconomic spectrum, this additional burden could be exacerbating existing health disparities for these already underserved groups. Therefore, future studies should further investigate cost as a structural barrier to naloxone use for uninsured, as well as individuals with coverage through state-based programs (Medicaid) or commercial payers. Additionally, the impact of state-level policies to reduce its cost for uninsured individuals, as providing naloxone to people who use opioids has been shown to be a cost-effective practice [44].

Limitations of this study include the lack of information gathered from each pharmacy. Callers did not collect information about the pharmacists themselves in terms of their age, gender, or experience working as a pharmacist in a independent community environment. Age or time since graduation may affect the results, as recently graduated pharmacists are more likely to have had naloxone training as a part of their didactic curriculum. Recent guidance from the American Association of Colleges of Pharmacies emphasizes the importance of educating students to address the public health crisis of opioid abuse, including expanding naloxone awareness and use [45]. Callers also did not ask whether naloxone is generally stocked; it is possible that some stores normally stock it but were sold out on the day the call was placed. Also, callers only completed calls with one pharmacist, not every pharmacist on staff. It is possible that different pharmacists may have varying levels of awareness and knowledge regarding naloxone, naloxone education has been a consistent part of pharmacy curriculum for student pharmacists and state-level CE for practicing, licensed pharmacists. However,due to the secret shopper methodology, we deemed pharmacies to be “complete” with data collection once the call was completed. Furthermore, callers only asked about the specific form of naloxone, Narcan, the intranasal spray. The other forms of naloxone covered in Georgia’s standing order, including intramuscular injections and auto-injectors, were not part of the callers’ questions. Pharmacists may have only responded regarding the availability of the specific formulation of naloxone. However, some pharmacists would offer other forms of naloxone when asked about Narcan.

There are other factors impacting naloxone acquisition and distribution that are not considered in this study. In particular, the social stigma surrounding naloxone usage, people who use opioids, and opioid use disorder may prevent someone from trying to obtain naloxone for themselves or for emergencies for others [9, 10]. Additionally, media coverage influences the public opinion of naloxone [46]. Pharmacists’ perceptions of naloxone distribution may also be skewed, and would therefore directly impact the potential availability of naloxone at independent pharmacies. Furthermore, the depth and breadth of counseling provided by those respective pharmacists may be impacted.

Future studies should address these limitations to provide a more complete picture of the barriers to naloxone access. The pharmacists who indicated that a prescription was required were not using the publicly available state naloxone standing order. This may be due to a lack of awareness or the decisions to not use the standing order, which could be explored further. Previous work has highlighted the need for additional educational programs for pharmacists to inform them of state law and policies [47]. Future studies should also analyze how to more effectively educate pharmacists regarding naloxone use, and how distribution affects overall use and distribution in the state. A recent study from Texas showed the positive impact of student-led academic detailing on improving pharmacists’ willing to dispense naloxone and increasing naloxone availability from community pharmacies [48].

## Conclusions

Despite its limitations, this study provided the first insight into the availability of naloxone at all independent community pharmacies in Georgia after the standing order was issued. It is clear that the availability of naloxone in Georgia’s independent community pharmacies is limited. The finding that a majority of pharmacists at independent community pharmacies in Georgia were not using the publicly available state naloxone standing order presents a barrier to access and may also contribute to the infrequency of independent pharmacies stocking naloxone. Additionally, the low availability of naloxone and its high cost for uninsured individuals are significant structural barriers for reducing opioid-related mortality. These findings should motivate policymakers and healthcare professionals in Georgia to increase education regarding naloxone prescribing laws and practices at the community pharmacy level and improve the availability and affordability of naloxone in order to make it more readily available to the general public.

## Data Availability

The datasets used or analyzed during the current study are available from the corresponding author on reasonable request.

## Abbreviations

US: United States
